# Supplementary material for: Induction of Expandable Tissue-Specific Progenitor Cells from Human Pancreatic Tissue through Transient Expression of Defined Factors
Source: Mol Ther Methods Clin Dev. 2019 Jan 29;13:243–52. doi: 10.1016/j.omtm.2019.01.011 (PMC6383192; doi:10.1016/j.omtm.2019.01.011)
Supplement: Document S1. Figures S1–S3 and Table S1 [file mmc1.pdf]

OMTM, Volume *13*

Supplemental Information

Induction of Expandable Tissue-Specific Progenitor Cells from Human Pancreatic Tissue through Transient Expression of Defined Factors

Hirofumi Noguchi, Chika Miyagi-Shiohira, Yoshiki Nakashima, Takao Kinjo, Naoya Kobayashi, Issei Saitoh, Masami Watanabe, A. M. James Shapiro, and Tatsuya Kin

A

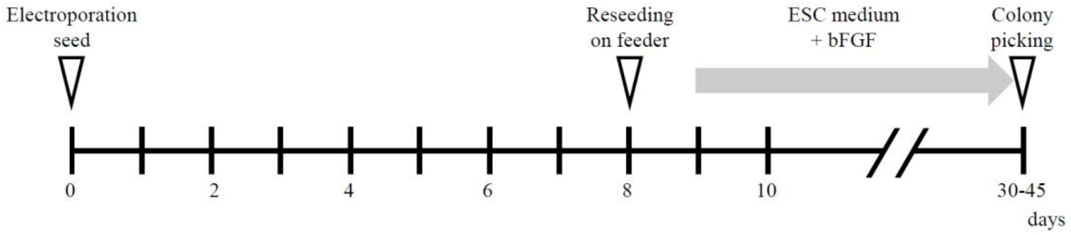

B

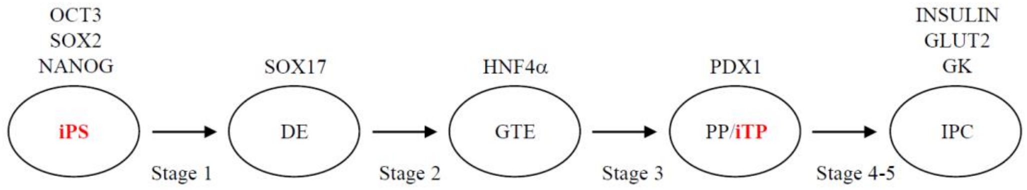

Supplemental Fig 1 Noguchi et al.

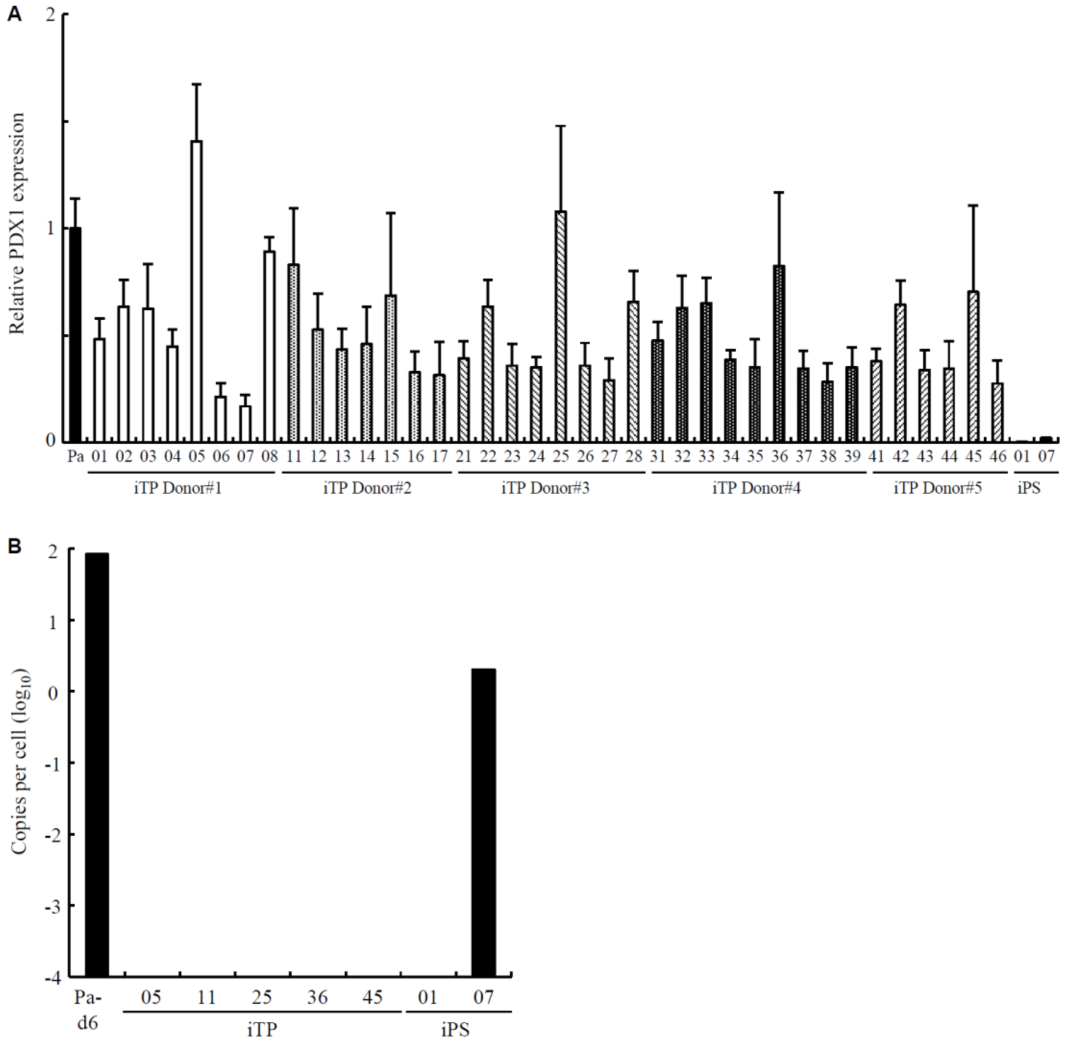

Supplemental Fig 2 Noguchi et al.

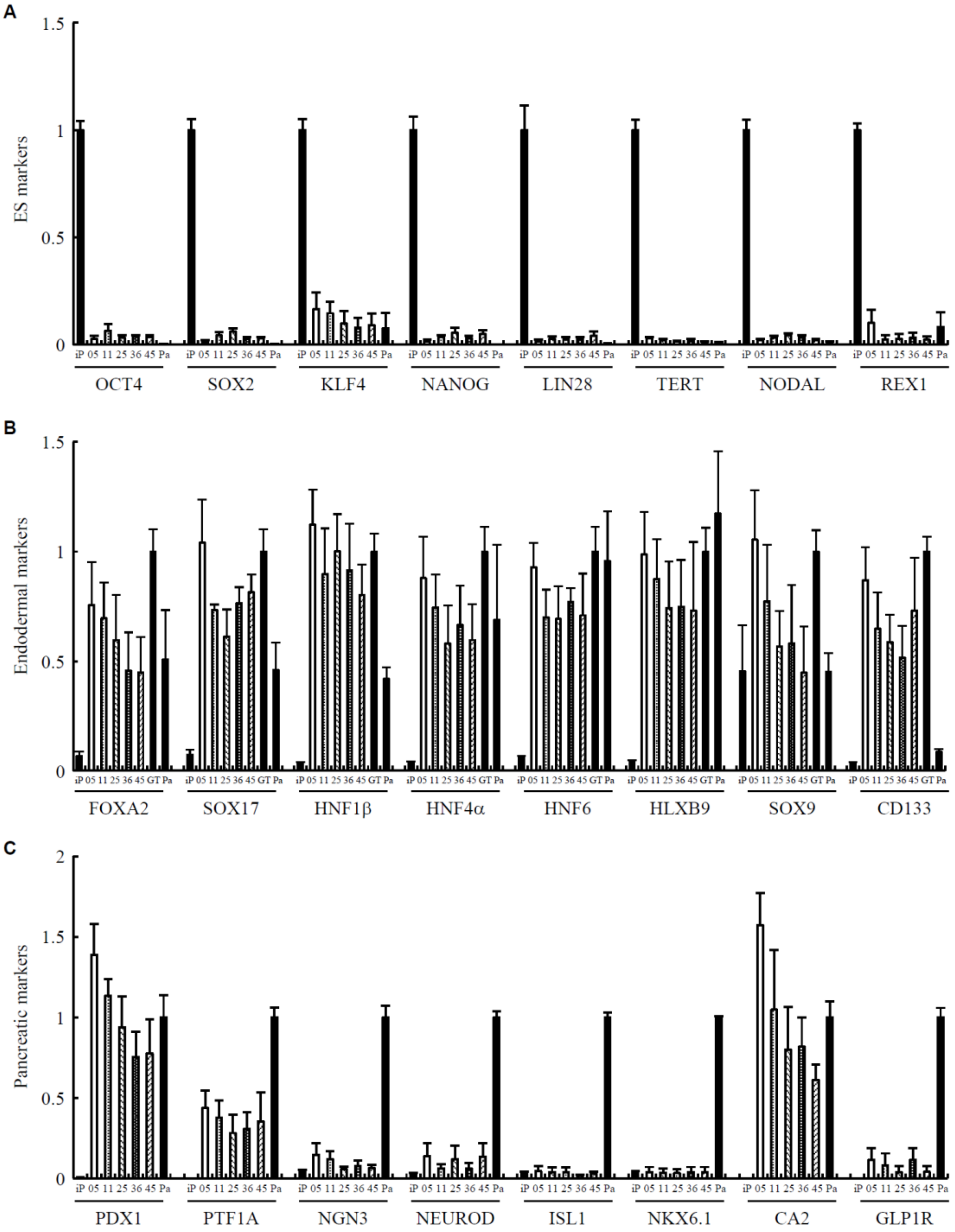

Supplemental Fig 3 Noguchi et al.

SUPPLEMENTAL FIGURE LEGENDS

Supplemental Figure 1. Time schedules for the generation of human iTP cells from pancreatic tissue and differentiation protocol of iPS/iTP cells. (A) Time schedules for the induction of iTP cells. Human pancreatic tissue (1×10^5 cells) was electroporated with episomal plasmid vectors expressing four (OCT3/4, SOX2, KLF4, c-MYC) or six (OCT3/4, p53 shRNA, SOX2, KLF4, L-MYC, and LIN28) reprogramming factors and plated on day 0. Cells were reseeded on feeder on day 8. Cells were then cultured in ESC medium plus bFGF. (B) A schematic representation of the stepwise differentiation of iPS/iTP cells to insulin-producing cells. The cells of the definitive endoderm (DE) express SOX17; cells of the gut tube endoderm (GTE) express HNF4α; pancreatic progenitors (PP) express PDX1; and insulin-producing cells (IPC) express INSULIN, GLUT2, and GLUCOKINASE (GK).

Supplemental Figure 2. Evaluation of human iTP cells from pancreatic tissue

(A) Quantitative RT-PCR analysis of PDX1, a marker of pancreatic stem/progenitor cells, in iTP and iPS cells. Thirty-eight iTP clones and two iPS clones were evaluated for PDX1 expression using quantitative RT-PCR. The data are expressed as the PDX1-to-GAPDH ratio, with the ratio of pancreatic tissue arbitrarily set to 1 (n = 5). Error bars

represent the standard error. (B) Copy numbers of episomal plasmid vectors in iTP and iPS clones. Pancreatic tissue 6 days after electroporation of plasmid vectors expressing six reprogramming factors were analyzed (Pa-d6) as a positive control.

Supplemental Figure 3. Quantitative RT-PCR analysis of human iTP cells for marker genes of ES and endodermal/pancreatic cells.

(A) qRT-PCR analysis of ES cell marker genes in human iTP05, 11, 25, 36, and 45 cells. iPS cells served as a control. (B) qRT-PCR analysis of endodermal cell marker genes in human iTP05, 11, 25, 36, and 45 cells. GTE cells were used as a control. (C) q RT-PCR analysis of pancreatic cell marker genes in human iTP05, 11, 25, 36, and 45 cells. Pancreatic cells (islets >80%) cells were used as a control. iP, iPS cells; 05/ 11/ 25/ 36/ 45, iTP cells; Pa, pancreatic cells (islets >80%); GT, GTE cells. The data are expressed as the gene-to-GAPDH ratio, with that of the control cells arbitrarily set to 1 (n = 4). The error bars represent the standard error.

Supplemental Table 1. Primers

	Forward	Reverse
OCT3/4 p	gaggttggagtagaaggattgttttggttt	cccccctaacccatcacctccaccacctaa
NANOG p	tggttaggttggttttaaatttttg	aacccacccttataaattctcaatta
PDX1 p	ctcagctgagagagaaaattggaa	actaatctcaaaaaaaacccac
