# Supplementary material for: Induction of Expandable Tissue-Specific Progenitor Cells from Human Pancreatic Tissue through Transient Expression of Defined Factors
Source: Mol Ther Methods Clin Dev. 2019 Jan 29;13:243–52. doi: 10.1016/j.omtm.2019.01.011 (PMC6383192; doi:10.1016/j.omtm.2019.01.011)
Supplement: Document S2. Article plus Supplemental Information [file mmc2.pdf]

Molecular Therapy
Methods & Clinical Development
Original Article

Induction of Expandable Tissue-Specific Progenitor Cells from Human Pancreatic Tissue through Transient Expression of Defined Factors

Hirofumi Noguchi,[1] Chika Miyagi-Shiohira,[1] Yoshiki Nakashima,[1] Takao Kinjo,[2] Naoya Kobayashi,[3] Issei Saitoh,[4] Masami Watanabe,[5] A. M. James Shapiro,[6] and Tatsuya Kin[6]

[1]Department of Regenerative Medicine, Graduate School of Medicine, University of the Ryukyus, Okinawa 903-0215, Japan; [2]Department of Basic Laboratory Sciences, School of Health Sciences in Faculty of Medicine, University of the Ryukyus, Okinawa 903-0215, Japan; [3]Okayama Saidaiji Hospital, Okayama 704-8192, Japan; [4]Division of Pediatric Dentistry, Graduate School of Medical and Dental Science, Niigata University, Niigata 951-8514, Japan; [5]Department of Urology, Okayama University Graduate School of Medicine, Dentistry and Pharmaceutical Sciences, Okayama 700-8558, Japan; [6]Clinical Islet Transplant Program and Department of Surgery, University of Alberta, Edmonton, AB, Canada

We recently demonstrated the generation of mouse induced tissue-specific stem (iTS) cells through transient overexpression of reprogramming factors combined with tissue-specific selection. Here we induced expandable tissue-specific progenitor (iTP) cells from human pancreatic tissue through transient expression of genes encoding the reprogramming factors OCT4 (octamer-binding transcription factor 4), p53 small hairpin RNA (shRNA), SOX2 (sex-determining region Y-box 2), KLF4 (Kruppel-like factor 4), L-MYC, and LIN28. Transfection of episomal plasmid vectors into human pancreatic tissue efficiently generated iTP cells expressing genetic markers of endoderm and pancreatic progenitors. The iTP cells differentiated into insulin-producing cells more efficiently than human induced pluripotent stem cells (iPSCs). iTP cells continued to proliferate faster than pancreatic tissue cells until days 100–120 (passages 15–20). iTP cells subcutaneously inoculated into immunodeficient mice did not form teratomas. Genomic bisulfite nucleotide sequence analysis demonstrated that the *OCT4* and *NANOG* promoters remained partially methylated in iTP cells. We compared the global gene expression profiles of iPSCs, iTP cells, and pancreatic cells (islets >80%). Microarray analyses revealed that the gene expression profiles of iTP cells were similar, but not identical, to those of iPSCs but different from those of pancreatic cells. The generation of human iTP cells may have important implications for the clinical application of stem/progenitor cells.

INTRODUCTION

Adult tissue-specific stem/progenitor cells present in multiple adult organs contribute to continuous tissue renewal or repair after injury and may therefore represent an alternative therapy for numerous diseases. Studies performed *in vitro* show that insulin (INS)-producing cells can be generated from adult pancreatic stem/progenitor cells.[1–3] The assessment of 83 human islet grafts transplanted using the Edmonton Protocol from 1999 to 2004[4] shows a significant positive correlation between the number of pancreatic progenitor (ductal-epithelial) cells transplanted and long-term metabolic success, which was assessed using an intravenous glucose tolerance test approximately 2 years after transplantation. Therefore, pancreatic duct/progenitor cells may serve as a new source of INS-producing cells.

In contrast, it is difficult to isolate pancreatic "stem" cells, which have unlimited self-renewal capacity. Although mouse pancreatic stem cell lines were established using specific culture conditions,[5,6] we could isolate such cells only from young mice.[7] Moreover, we were unable to isolate pancreatic stem cells from human pancreatic tissue.[8] The unlimited availability of normal tissue-specific stem/progenitor cells will undoubtedly contribute to a better understanding of stem cell biology that is critical for effective organ repopulation in the application of regenerative medicine. However, it is extremely difficult to purify or expand tissue-specific stem/progenitor cells from native tissues, because the population of such cells is very small.

Induced pluripotent stem cells (iPSCs), which are generated from adult fibroblasts or other somatic cells, are similar to embryonic stem cells (ESCs) in their morphology, gene expression pattern, epigenetic status, and ability to differentiate into cells derived from the three embryonic germ layers.[9–15] iPSCs can be generated without the genomic integration of genes encoding exogenous reprogramming factors carried by plasmids,[16–18] adenoviruses,[19] or synthetic RNAs.[20] Moreover, the production of iPSCs without insertional mutagenesis addresses a critical safety concern for their potential use in regenerative medicine. However, the clinical application of iPSCs is hampered by their ability to form teratomas and their limited

Received 19 September 2018; accepted 17 January 2019;
https://doi.org/10.1016/j.omtm.2019.01.011.

Correspondence: Hirofumi Noguchi, MD, PhD, Department of Regenerative Medicine, Graduate School of Medicine, University of the Ryukyus, 207 Uehara, Nishihara, Okinawa 903-0215, Japan.
E-mail: noguchih@med.u-ryukyu.ac.jp

This is an open access article under the CC BY license (http://creativecommons.org/licenses/by/4.0/).

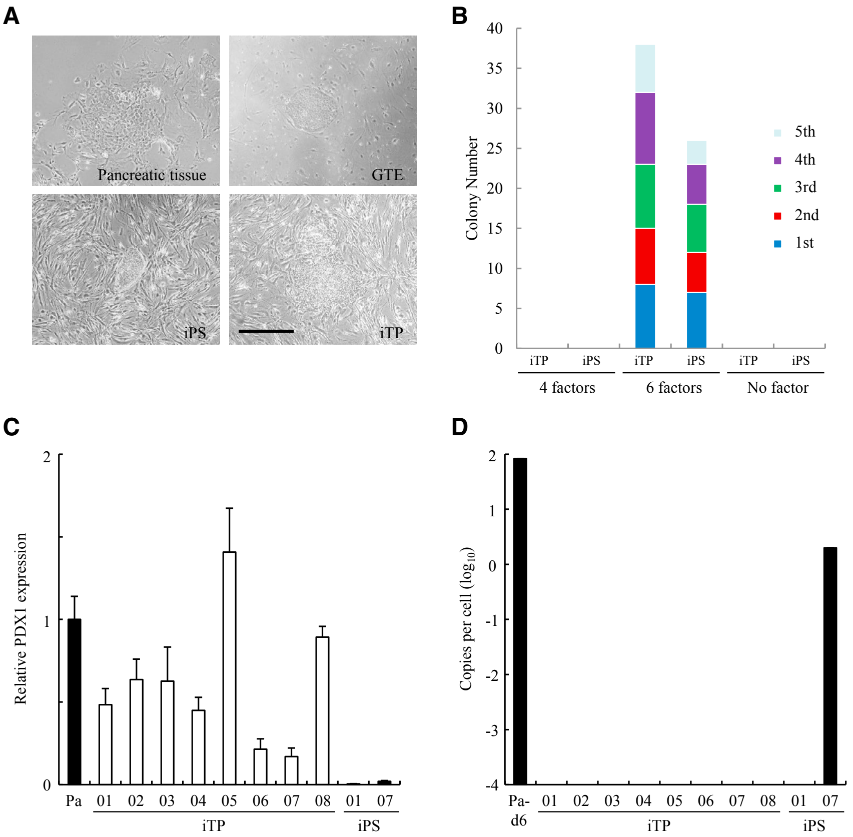

Figure 1. Generation of Human iTP Cells from Pancreatic Tissue

(A) The morphologies of human pancreatic tissue, GTE cells, iPSCs, and iTP cells. Scale bar, 200 μm. (B) Numbers of colonies of iTP and iPSCs. Episomal plasmid vectors were transfected into human pancreatic tissue, and the number of colonies was counted after 30–45 days. (C) qRT-PCR analysis of PDX1, a marker of pancreatic stem/progenitor cells, in iTP and iPSCs. Eight iTP clones and two iPS clones were evaluated for PDX1 expression using qRT-PCR. The data are expressed as the PDX1-to-GAPDH ratio, with the ratio of pancreatic tissue arbitrarily set to 1 (n = 5). Error bars represent the SE. (D) Copy numbers of episomal plasmid vectors in iTP and iPS clones. Pancreatic tissue 6 days after electroporation of plasmid vectors expressing six reprogramming factors were analyzed (Pa-d6) as a positive control.

potential to generate pure populations of differentiated cell types *in vitro*.

Recently, we focused on developing a method for generating induced tissue-specific/progenitor stem (iTS/iTP) cells by transfecting cells with a plasmid harboring cDNAs encoding octamer-binding transcription factor (OCT) 3/4, sex-determining region Y-box (SOX) 2, Kruppel-like factor 4 (KLF4), and MYC, followed by tissue-specific selection.[21–24] The iTS cells derived from mouse pancreas (iTS-P) or liver (ITS-L), which express several genetic markers for endoderm and pancreatic/hepatic progenitors, differentiated into INS-producing cells/hepatocytes more frequently than ESCs upon the induction of differentiation. More important, the iTS-P/iTS-L cells were unable to generate teratomas when subcutaneously transplanted into immunodeficient mice. Moreover, evidence indicates that after the reprogramming of mouse/human iPSCs, epigenetic memory is inherited from the parental cells, and the iPSCs with epigenetic memory differ from ESCs in their gene expression profiles, persistence of donor cell gene expression, and ability to differentiate.[25–30] Therefore, iTS cells inherit numerous components of epigenetic memory from pancreas/liver cells and acquire self-renewal potential.

Here we generated expandable iTP cells from human pancreatic tissue using episomal plasmid vectors expressing OCT4, p53 small hairpin RNA (shRNA), SOX2, KLF4, L-MYC, and LIN28.

RESULTS

Generation of Human iTP Cells from Pancreatic Tissue

We attempted to generate human iTP cells from pancreatic tissue (>80% islets) by transfection of episomal plasmid vectors expressing the reprogramming factors OCT4, SOX2, KLF4, and MYC or OCT4, p53 shRNA, SOX2, KLF4, L-MYC, and LIN28.[17] We generated 64 colonies (Figure 1A) using the latter set of reprogramming factors (Figure 1B). Of the 64 clones, 26 showed an iPS-like morphology and generated teratomas (Table 1). The other 38 clones exhibited an iTP-like morphology similar to that of gut tube endodermal (GTE) cells. GTE cells were generated using a stepwise differentiation protocol that relied on intermediates thought to be similar to the cell populations present in the developing embryo.[31,32] The latter 38 clones did not generate teratomas (Figure 1A; Table 1). Eight of the latter clones were evaluated for their expression of pancreatic and duodenal homeobox factor (PDX) 1, a marker of pancreatic stem/progenitor cells. All clones expressed *PDX1* mRNA (Figure 1C).

We next estimated the copy numbers of the episomal plasmid vectors in these clones using a PCR primer pair to amplify the *EBNA-1* sequence of Epstein-Barr virus.[17] Approximately 100 copies of the episomal plasmid vectors per cell were detected 6 days after transfection. In contrast, *EBNA-1* DNA was undetectable in eight clones tested at passage 10. One of two iPS clones contained two copies, indicating chromosomal integration of the plasmid (Figure 1D). We used clone iTP05 for subsequent experiments because it expressed the highest levels of *PDX1* mRNA.

Genes of Interest Expressed by Human iTP Cells

ESC marker genes expressed by iTP05 cells were detected using RT-PCR assays. The levels of mRNAs encoding the pluripotency markers such as OCT4, SOX2, and NANOG were significantly lower

Table 1. Teratoma Formation

Cell Type	Injected Cell Number	Mice Bearing Teratoma/Total Mice Injected	Period (Days)
iPS	1×10^6	26/26	60
iTP	1×10^6	0/38	150

compared with those of iPSCs (Figure 2A). We next investigated the expression patterns of genes encoding endodermal markers. GTE cells generated from iPSCs were used as a positive control. The expression of endodermal marker genes such as forkhead box protein a2 (FOXA2) and hepatocyte nuclear factors 1β, 4α, 6 (HNF1β, 4α, 6) was detected in iTP05 cells (Figure 2B) in a pattern similar to that of GTE cells, but not iPSCs. We next investigated the gene expression patterns of pancreatic markers. Pancreatic tissues (>80% islets) were used as a positive control. The expression of PDX1, PTF1A, and CA2 was detected in iTP05 cells, and NEUROD, ILS1, and NKX6.1 were expressed at lower levels (Figure 2C).

Proliferation of Human iTP Cells

We previously found that human pancreatic progenitor cells (duct-rich population) proliferate until day 30.[8] Here we evaluated the proliferation of human pancreatic tissue cells, iTP cells, and iPSCs. Human pancreatic tissue cells (including pancreatic duct cells) divided until day 30. iTP cells continued to divide faster than pancreatic tissue cells after day 30 without detectable changes in their morphology or proliferation rate. However, iTP cells stopped dividing on days 100–120 (passages 15–20). Therefore, the cells were "progenitor" cells rather than "stem" cells with unlimited self-renewal capability. The iPSCs proliferated after day 120 without detectable changes in their morphology or proliferation rate (Figure 3A).

Differentiation of Human iTP Cells into INS-Producing Cells

To evaluate the potential of iTP05 cells to differentiate, we applied a stepwise differentiation protocol.[31,32] iTP05 cells express endodermal cell markers. Therefore, we included stages 4 and 5 of the induction protocol of the stepwise differentiation protocol. iTP05 cells expressed INS and its mRNA more efficiently than iPSCs (Figures 3B and 3C). The INS-positive cells were C-PEPTIDE-positive, thus excluding the possibility of INS uptake from the medium, and 15.4% ± 1.8% of the differentiated cells were INS/C-PEPTIDE-double positive.

To determine the glucose sensitivity of the cells differentiated from the iTP05 clone, we exposed them to low (2.8 mM) and then high (20 mM) concentrations of glucose. The cells released approximately 2- to 3-fold higher amounts of human INS than an iPS-derived population in the presence of both glucose concentrations (Figure 3D). The stimulation index of the cells differentiated from iTP05 cells was higher compared with that of the iPSCs, although the difference was not statistically significant (Figure 3E).

Differentiated iTP cells were transplanted into nude mice. The graft contained approximately 15% INS-positive cells (Figure 3F, right panel). In contrast, Ki67-positive cells represented <5% of the engrafted cells (Figure 3F, left and middle panels), suggesting that differentiated iTP cells infrequently proliferated after transplantation.

Bisulfite Genomic Sequencing of the Promoter Regions of *OCT4* and *NANOG* in iTP and iPS Cells

Bisulfite genomic nucleotide sequencing demonstrated that the *OCT4* and *NANOG* promoters remained methylated in iTP cells but were demethylated in iPSCs. In contrast, the *PDX1* promoters were demethylated in iTP cells (Figure 4). These results demonstrate that methylation of these promoters in iTP cells differs from that in iPSCs.

Microarray Analysis

We performed microarray analysis to compare the global gene expression profiles of human iPSCs, iTP cells, and pancreatic tissue cells (islets >80%). Of 54,613 genes, the levels of 7.6% differed by >2-fold between iPSCs and iTP cells; the levels of 9.9% were >2-fold different between pancreatic tissue and iTP cells; and the levels of 16.5% were >2-fold different between iPSCs and pancreatic tissue (Figure 5A). These data suggest that the expression pattern of iTP cells was similar to that of iPSCs but somewhat different from that of pancreatic tissue. Unsupervised hierarchical clustering of gene expression profiles of iPSCs, iTP cells, and pancreatic tissue showed that iTP cells clustered more closely with iPSCs than pancreatic tissue cells (Figure 5B), although the phenotypes of iTP cells markedly differed from those of iPSCs.

Restriction of the Developmental Potential of iTP Cells

To determine whether the developmental potential of human iTP cells was restricted to pancreatic lineages, cultures were induced using the conditions established to drive iPSCs toward hepatocytes,[33] neuroectoderm,[34] or mesoderm.[33] Increased levels of mRNAs encoding the liver markers ALBUMIN (ALB) or α1-AT (Figures 6A and 6B), the mesodermal markers platelet/endothelial cell adhesion molecule 1 (PECAM1), or Mix1 homeobox-like 1 (MIXL1) (Figures 6C and 6D), as well as those of the neuroectodermal markers zinc-finger protein of the cerebellum 1 (ZIC1) or SOX1 (Figures 6E and 6F), were not detectable in the iTP cells, suggesting that iTP cells are committed to tissue-specific differentiation.

Reproducible Generation of Human iTP Cells

We investigated the characteristics of human iTP cells derived from each of five human donors to evaluate the reproducibility of generating iTP cells. Thirty-eight clones that exhibited an iTP-like morphology expressed *PDX1* mRNA (Figure S2A). The five clones that expressed the highest levels of *PDX1* mRNA, iTP05, iTP11, iTP25, iTP36, and iTP45, were selected for further analysis. PCR analysis did not detect *EBNA-1* DNA in any of the clones after passage 10 (Figure S2B).

We next used RT-PCR to detect the expression of genes that serve as markers of ESCs, endodermal cells, or pancreatic cells. The levels of mRNAs encoding pluripotency markers such as OCT4, SOX2, and NANOG were significantly lower compared with those of iPSCs (Figure S3A). The pattern of expression of endodermal marker genes such as FOXA2 and HNF1β, 4α, 6 (Figure S3B) was similar to that of GTE

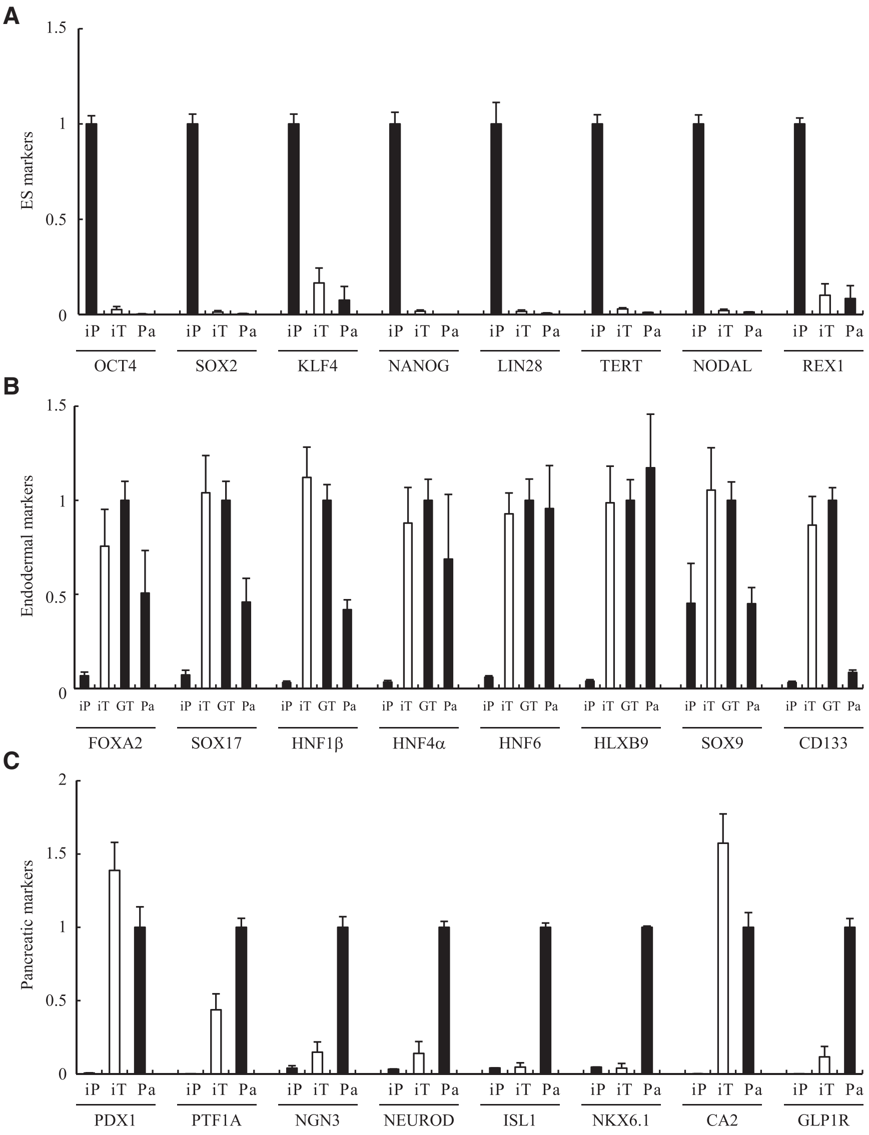

Figure 2. qRT-PCR Analysis of Human iTP Cells for Marker Genes of ESCs and Endodermal/Pancreatic Cells

(A) qRT-PCR analysis of ESC marker genes in human iTP05 cells. iPSCs served as a control. (B) qRT-PCR analysis of endodermal cell marker genes in human iTP05 cells. GTE cells were used as a control. (C) qRT-PCR analysis of pancreatic cell marker genes in human iTP05 cells. Pancreatic cells (islets >80%) were used as a control. The data are expressed as the gene-to-GAPDH ratio, with that of the control cells arbitrarily set to 1 (n = 4). The error bars represent the SE. GT, GTE cells; iP, iPSCs; iT, iTP05 cells; Pa, pancreatic cells (islets >80%).

cells, but not iPSCs. Each clone expressed mRNAs encoding PDX1, PTF1A, and CA2 and lower levels of mRNAs encoding NEUROD, ILS1, and NKX6.1 (Figure S3C). These data suggest that it is possible to reproducibly generate iTP cells.

DISCUSSION

Here we show that enforced transient expression of six reprogramming factors in differentiated pancreatic cells induced the generation of iTP cells with functional and molecular attributes corresponding to their tissue-specific progenitor cells. Further, the iTP cells were readily expanded *in vitro*. Although mouse pancreatic stem cells have been identified,[5–7] it is extremely difficult to isolate human pancreatic stem cells capable of self-renewal.[8] Therefore, the generation of human iTP cells using iPS cell technology may contribute to the development of new treatments for diabetes.

We show here that the self-renewal capacity of human iTP cells was significantly higher than that of normal pancreatic progenitor cells (duct-rich population), although normal pancreatic progenitor cells and iTP cells have limited self-renewal capacity (Figure 3A). In contrast, we previously reported the generation of mouse iTS-P cells with unlimited self-renewal capacity.[6] The difference in the self-renewal capacity between human and mouse cells may be explained by differences in epigenetic alterations during reprogramming. We previously generated mouse iTS-P cells using four reprogramming factors in our previous study.[6] Here we generated human iTP cells using six reprogramming factors. Similarly, mouse iPSCs are generated using plasmids expressing four factors,[16] whereas human iPSCs are generated using plasmids expressing six factors.[17,18] Thus, epigenetic changes during the reprograming of mouse cells may be less complex than those during the reprograming of human cells.

The difference in the self-renewal capacity between human and mouse cells may be further explained by the differences in the conditions used to culture human and mouse ESCs. Although the culture conditions for mouse ESCs (culture media containing leukemia inhibitory factor) are suitable for mouse pancreatic stem cells and iTS-P cells, it is unclear whether those for human ESCs (culture media containing basic fibroblast growth factor [bFGF]) are suitable for human pancreatic stem cells and iTS-P cells. Thus, if we generate iTS-P cells from human pancreatic tissue, the cells may not maintain the undifferentiated phenotype when cultured under unsuitable conditions.

The amount of INS secreted by iTP cells was higher compared with that of iPSCs. However, the absolute value of the stimulation index was quite low, suggesting that INS-producing cells generated from iTP cells were unable to fully respond to fluctuations in glucose concentrations. To our knowledge, there is no established protocol for

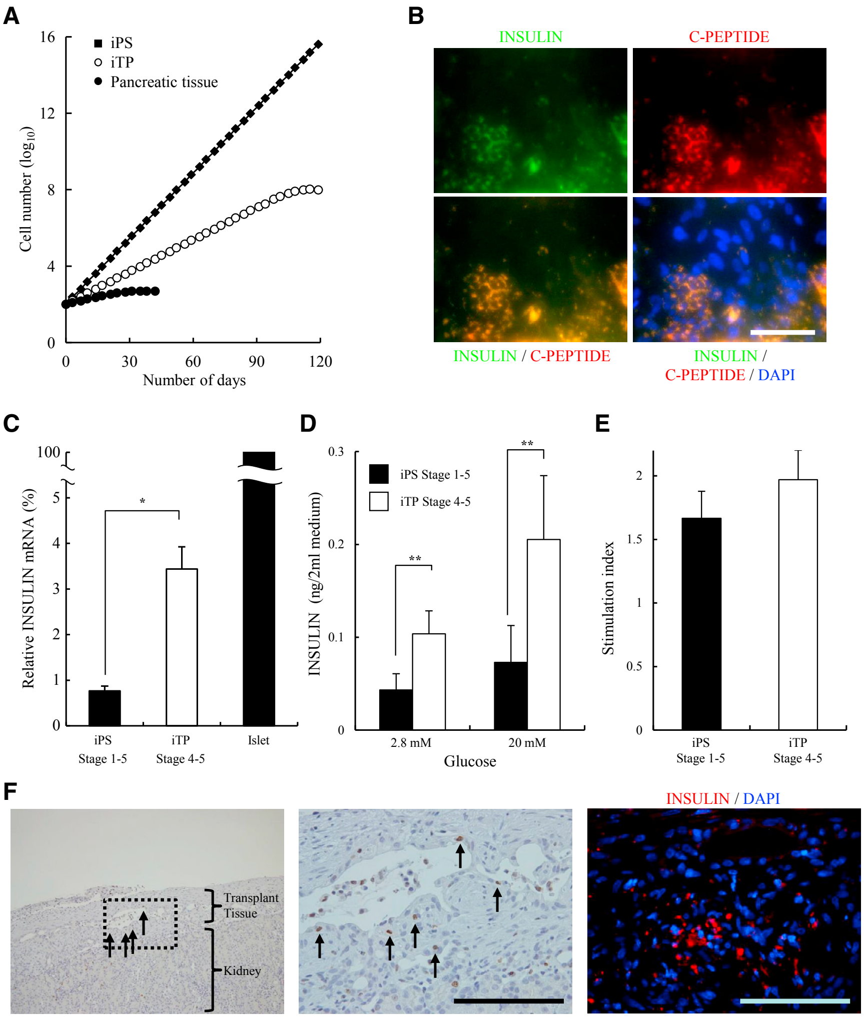

Figure 3. Proliferation of iTP Cells and Their Differentiation into Insulin-Producing Cells

(A) Growth curves of iPSCs, iTP cells, and pancreatic tissue cells. (B) Immunohistochemical analysis of INS-producing cells (INS, C-PEPTIDE) derived from iTP05 cells treated with the stepwise protocol. Scale bar, 100 μm. (C) qRT-PCR analysis of INS expression in differentiated iTP05 cells. qRT-PCR analysis of differentiated cells derived from iTP05 cells (passage 10) at stages 4–5, derived from iPSCs at stages 1–5. Isolated islets (islets >80%) were used as a positive control. The data are expressed as the INS:GAPDH ratio, with that of the islets arbitrarily set to 100 (n = 4). Error bars represent the SE. (D) INS release assay. Differentiated cells derived from iTP05 cells (passage 10) using the stage 4–5 protocol and derived from iPSCs using the stage 1–5 protocol were treated with 2.8 and 20 mM D-glucose, and the amount of INS released into the culture supernatant was analyzed using an ELISA. Error bars represent the SE. (E) The stimulation index shown in (D). Error bars represent the SE. *$p < 0.05$. (F) Immunohistochemical analysis of Ki67 expression (left panel, low magnification; middle panel, high magnification [dotted square, left panel] and INS expression (right panel, red cells) derived from engrafted iTP05 cells in the graft. Scale bars, 100 μm.

inducing the differentiation of pancreatic stem/progenitor cells into INS-producing cells. When we transplanted differentiated iTP cells into nude mice with diabetes, the mice did not become normoglycemic. Establishing an efficient, reproducible protocol for generating INS-producing cells is critically important for clinical applications.

We show here that human iTP cells differentiated into INS-producing cells more efficiently than iPSCs and did not form teratomas. In striking contrast, ESCs/iPSCs may form teratomas, even after transplantation of differentiated cells derived from ESCs/iPSCs, because of possible contamination with undifferentiated cells. The decreased potential for teratoma formation illustrates an advantage of using iTP cells for regenerative medicine compared with ESCs/iPSCs. Further, bisulfite nucleotide sequence analysis of genomic DNA performed here clearly demonstrates that the promoters of *OCT4* and *NANOG* remained methylated in iTP cells, whereas the promoters were demethylated in iPSCs. Moreover, qRT-PCR assays detected low levels of *OCT4* or *NANOG* mRNA. These results demonstrate that the methylation of these promoters in iTP cells was not similar to that of iPSCs.

The global gene expression profiles of iPSCs, iTP cells, and pancreatic tissue cells show that iTP cells markedly differed from iPS and pancreatic cells. Unsupervised hierarchical clustering of gene expression profiles shows that iTP cells clustered more closely with iPSCs than pancreatic cells (Figure 5). Thus, the expression profile and genomic methylation status of iTP cells clearly differed from those of iPSCs and pancreatic islets.

In conclusion, we generated human iTP cells from pancreatic cells using episomal plasmid vectors expressing six reprogramming factors. Another group recently generated expandable induced tissue-specific stem/progenitor cells with characteristics similar to those of the iTS/iTP cells studied here, through the transient expression of YAP/TAZ,[35] as well as with endodermal stem/progenitor cells using defined small molecules.[36] iTS/iTP cells provide advantages over iPSCs. For example, they are easier to generate, differentiate efficiently, and do not form teratomas. The regeneration of pancreatic-β cells from stem and progenitor cells is an attractive method for restoring the islet cell mass. We believe that our present findings provide compelling evidence that our protocol for inducing tissue-specific stem/progenitor cells using reprogramming factors will advance the field of regenerative medicine.

MATERIALS AND METHODS

Generation of iPSCs and iTP Cells from Human Pancreatic Cells

Pancreatic cells (>80% islets) from human neurological determination of death (NDD) donors (woman, age 20–40 years, 5 cases) were isolated at the University of Alberta after donor family and human ethics

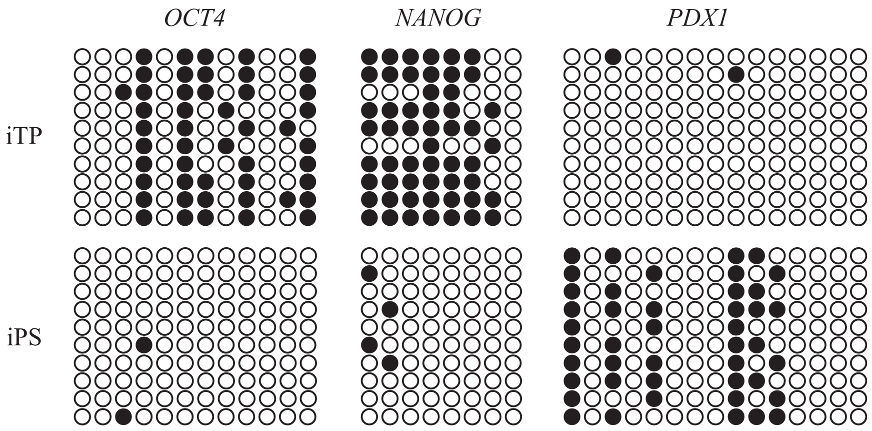

Figure 4. Bisulfite Sequence Analysis of Genomic DNA

Bisulfite sequence analysis of the *OCT4*, *NANOG*, and *PDX1* promoter regions in iTP05 cells and iPSCs. Open and closed circles indicate unmethylated and methylated CpG dinucleotides, respectively.

research consents were obtained, as previously described.[37] Cells were shipped to Japan and maintained in DMEM (Life Technologies, Carlsbad, CA, USA) containing 10% fetal bovine serum (FBS; Thermo Scientific, Kanagawa, Japan) and 0.5% penicillin (Sigma-Aldrich, St. Louis, MO, USA). Plasmid vectors expressing the reprogramming factors OCT4, SOX2, KLF4, MYC or OCT4, p53 shRNA, SOX2, KLF4, L-MYC, and LIN28[17] were electroporated into 6×10^5 pancreatic cells using a Microporator (Invitrogen) with a 100-μL kit according to the manufacturer's instructions. For pancreatic cells, conditions were 1,650 V, 10 ms, and three pulses. The cells were trypsinized 7 days after transfection, and 1×10^5 cells were replated onto 100-mm dishes covered with a mouse embryo fibroblast (MEF) feeder layer. The culture medium was replaced the next day with primate ESC medium containing bFGF (Repro CELL, Kanagawa, Japan). The colonies were counted 30–45 days after plating, and colonies similar to human ESCs or GTE cells were selected for further cultivation and evaluation (Figure S1A).

Cell Culture

iPS and iTP cells (induced cells described above) were maintained on an MEF feeder layer in DMEM-F12 (Sigma-Aldrich), 2 mM L-glutamine (Nacalai Tesque, Kyoto, Japan), 1:100 dilution of nonessential amino acids (Life Technologies), 0.1 mM β-mercaptoethanol (Sigma-Aldrich), 5 ng/mL bFGF (Repro CELL), and penicillin/streptomycin (Sigma-Aldrich). For passaging, iPS/iTP colonies were dissociated with Dissociation Solution for human ESCs/iPSCs (Riken CDB, Kobe, Japan) and split at ratios between 1:3 and 1:6.

Teratoma Formation/Tumorigenicity Assay

All mouse studies were approved by the Review Committee of the University of the Ryukyus. Eight-week-old non-obese diabetic/severe combined immunodeficiency (NOD/SCID) mice (CLEA, Tokyo, Japan) were used for teratoma formation studies. iPS/iTP cells (1×10^6) were inoculated into the humerus and thigh of NOD/SCID mice.

qRT-PCR

Total RNA was extracted from cells using an RNeasy Mini Kit (QIAGEN, Tokyo, Japan). After quantifying the RNA by spectrophotometry, 2.5 μg of RNA was heated at 85°C for 3 min and then reverse-transcribed in a 25-μL solution containing 200 U of Superscript II RNase H-RT (Invitrogen), 50 ng of random hexamers (Invitrogen), 160 μmol/L dNTP, and 10 nmol/L dithiothreitol. The reactions were incubated for 10 min at 25°C, 60 min at 42°C, and 10 min at 95°C. mRNAs were quantified using a TaqMan real-time PCR system according to the manufacturer's instructions (Applied Biosystems, Foster City, CA, USA). PCR was performed for 40 cycles, including 2 min at 50°C and 10 min at 95°C as initial steps. In each cycle, denaturation was performed for 15 s at 95°C, and annealing/extension was performed for 1 min at 60°C. PCR was performed in a 20-μL solution containing cDNAs synthesized from 1.11 ng of total RNA. For each sample, the levels of mRNAs were normalized by dividing by them by the levels of GAPDH. Primers for human genes encoding OCT4, SOX2, KLF4, NANOG, LIN28, TERT, NODAL, REX, FOXA2, SOX17, HNF1β, HNF4α, HNF6, HLXB9, SOX9, CD133, PDX1, PTF1a, NGN3, NEUROD, ISL1, NKX6.1, CA2, GLP1R, INS, ALB, α1-AT, PECAM1, MIXL1, ZIC1, SOX1, and GAPDH were purchased from Assays-on-Demand Gene Expression Products (Applied Biosystems). A PCR primer pair representing the *EBNA-1* sequence derived from Epstein-Barr virus[17] was used to estimate the copy numbers of episomal plasmid vectors.

Cell Induction and Differentiation

Directed differentiation into INS-producing cells was conducted as described previously,[31,32] with minor modifications. iPSCs (passage 10) and iTP cells (passage 10) were used in this experiment. In stage 1, cells were treated with 25 ng/mL Wnt3a and 100 ng/mL activin A (R&D Systems, Minneapolis, MN, USA) in RPMI (Invitrogen) for 1 day, followed by treatment with 100 ng/mL activin A in RPMI + 0.2% FBS for 2 days. In stage 2, the cells were treated with 50 ng/mL FGF10 (R&D Systems) and 0.25 μM 3-keto-N-(aminoethyl-aminocaproyl-dihydrocinnamoyl) (KAAD)-cyclopamine (Toronto Research Chemicals, Toronto, Canada) in RPMI + 2% FBS for 3 days. In stage 3, the cells were treated with 50 ng/mL FGF10, 0.25 μM KAAD-cyclopamine, and 2 μM all-*trans* retinoic acid (Sigma-Aldrich) in DMEM + 1% (v/v) B27 supplement (Invitrogen) for 3 days. In stage 4, the cells were treated with 1 μM N-[N-(3, 5-difluorophenacetyl)-1-alanyl]-S-phenylglycinet-butylester (DAPT; Sigma-Aldrich) and 50 ng/mL exendin-4 (Sigma-Aldrich) in DMEM + 1% (v/v) B27 supplement for 3 days. In stage 5, the cells were treated with 50 ng/mL exendin-4, 50 ng/mL IGF-1 (Sigma-Aldrich), and 50 ng/mL hepatocyte growth factor (R&D Systems) in Connaught Medical Research

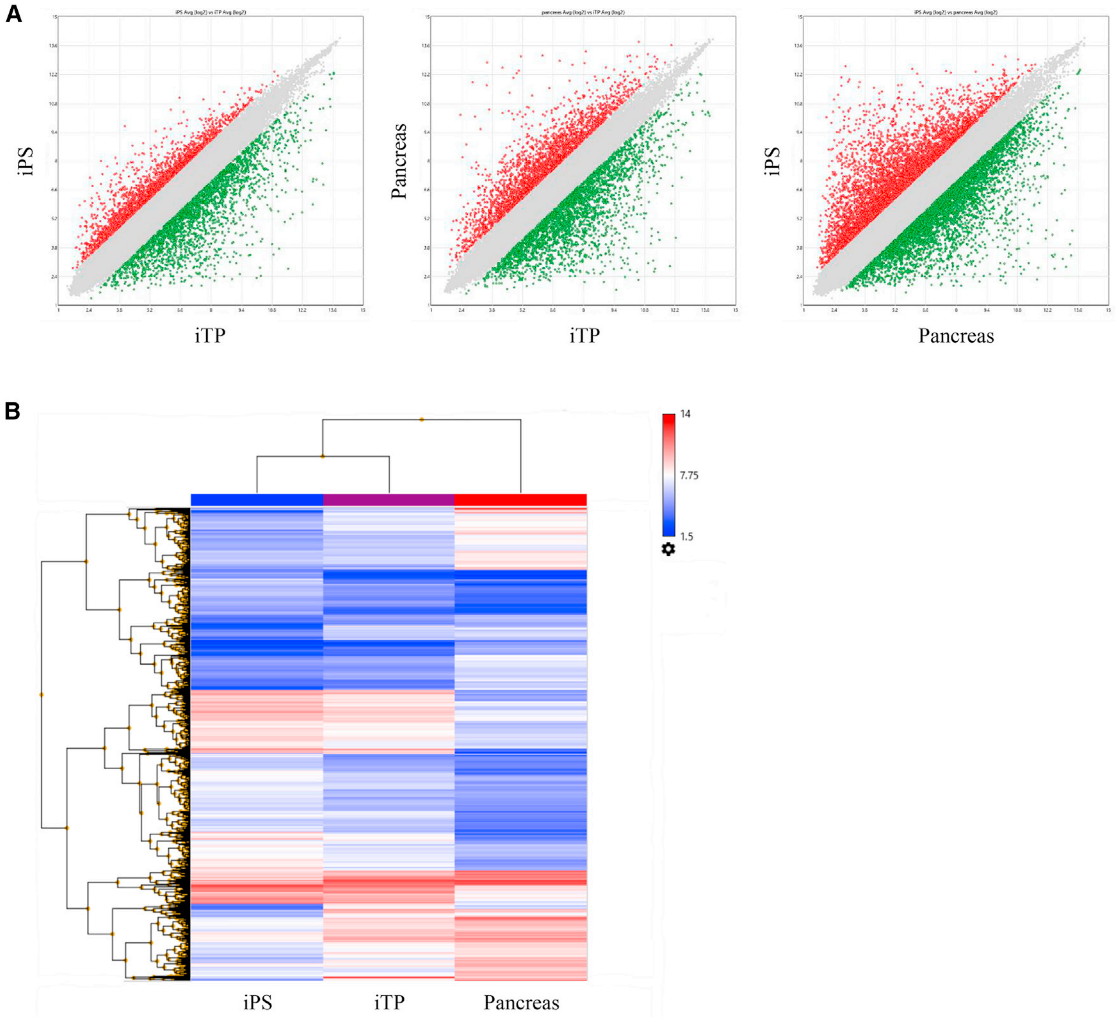

Figure 5. Microarray Analysis

(A) Global gene expression patterns were compared between iPSCs and iTP cells, between pancreatic tissue cells and iTP cells, and between iPSCs and pancreatic tissue cells using a Transcriptome Analysis Console (Affymetrix). The gray area indicates genes expressed at levels <2-fold different between the two samples. (B) Unsupervised hierarchical clustering of gene expression profiles of iPSCs, iTP cells, and pancreatic tissue cells. Each column represents one biological sample.

Laboratories medium (CMRL; Invitrogen) + 1% (v/v) B27 supplement for 3–6 days (Figure S1B).

Immunohistochemistry

The cells were fixed with 4% paraformaldehyde in PBS. After blocking with 20% AquaBlock (EastCoast Bio, North Berwick, ME, USA) for 30 min at room temperature, the cells were incubated overnight at 4°C with a guinea pig anti-INS antibody (1:100; Abcam, Tokyo, Japan) or rabbit anti-C-PEPTIDE antibody (1:200; Cell Signaling Technology, Danvers, MA, USA) and then for 1 h at room temperature with fluorescein isothiocyanate (FITC) or Alexa Fluor 647-conjugated anti-guinea pig immunoglobulin G (IgG) (FITC, 1:250 [Abcam] and Alexa Fluor 647, 1:250 [Cell Signaling Technology]), or Alexa Fluor 647-conjugated anti-rabbit IgG (1:250; Cell Signaling Technology). The cells were mounted on slides using VECTASHIELD Antifade Mounting Medium with DAPI (Vector Laboratories, Peterborough, UK). The percentage of INS/C-PEPTIDE-positive cells was calculated based on the ratio of immunostaining-positive cells/DAPI-positive cells in 10 visual fields.

To identify proliferating cells, we used immunohistochemistry (IHC) to detect Ki67 in the nuclei of cells in the G1, S, G2, and M phases of the cell cycle. For this purpose, we used the Histofine Simple Stain MAX PO (R) kit (Nichire Biosciences, Tokyo, Japan) with an anti-Ki67 antibody (ab 15580) (Abcam, Cambridge, UK).

Transplantation of Differentiated iTP Cells

Differentiated iTP cells were transplanted into the renal subcapsular space of the left kidneys of nude mice. One week after transplantation,

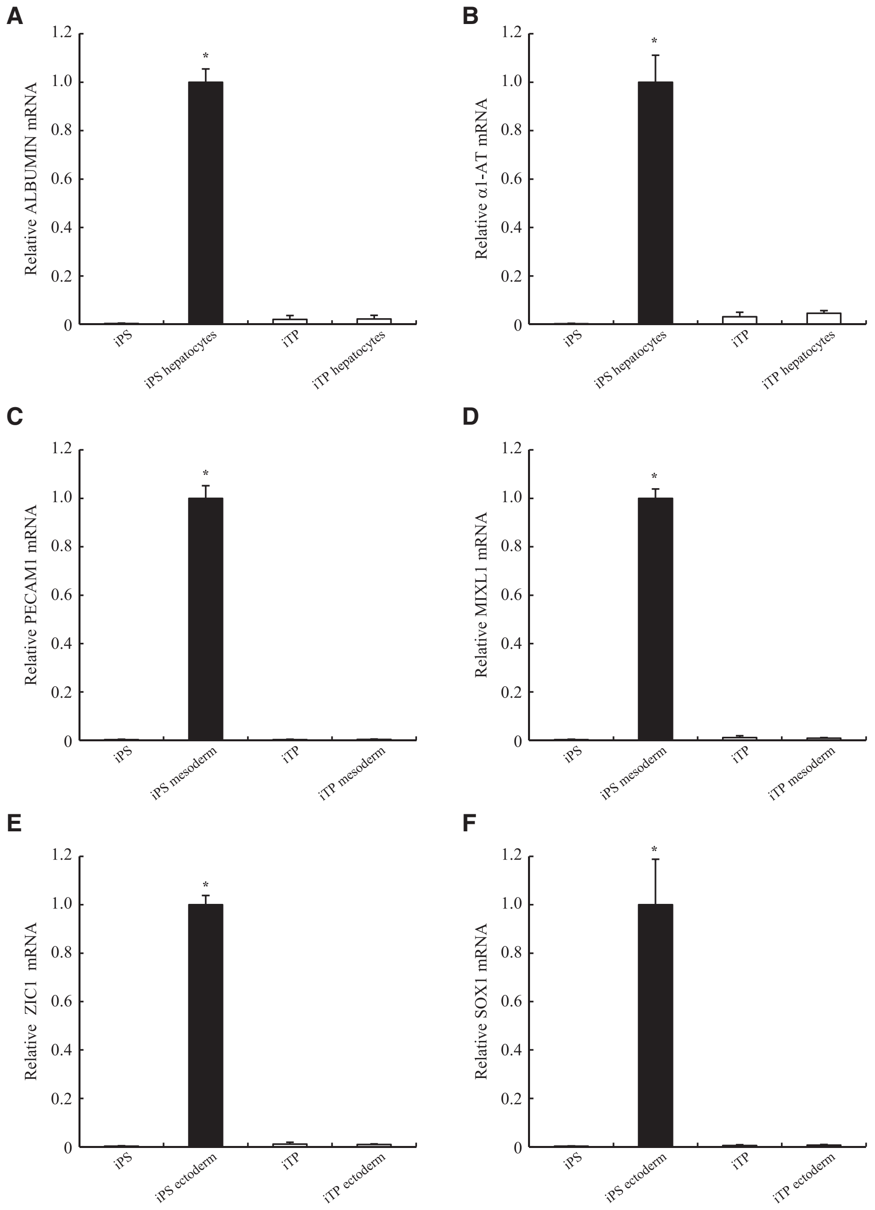

Figure 6. Restriction of the Developmental Potential of iTP Cells

(A) qRT-PCR analysis of ALB expression in undifferentiated and differentiated iTP cells. (B) qRT-PCR analysis of α1-AT in undifferentiated and differentiated iTP cells. (C and D) qRT-PCR analysis of mesodermal genes PECAM1 (C) and MIXL1 (D) in undifferentiated and differentiated iTP cells. (E and F) qRT-PCR analysis of the expression of the neuroectodermal genes ZIC1 (E) and SOX1 (F) in undifferentiated and differentiated iTP cells. iPSCs and the differentiated cells derived from iPSCs were used as controls. *$p < 0.05$ compared with other cells. The data are expressed as the gene-to-GAPDH ratio, with that of the differentiated cells from iPSCs arbitrarily set to 1 (n = 4). Error bars represent the SE.

the grafts were harvested and subjected to IHC using antibodies against Ki67 and INS. Studies using mice were approved by the Institutional Animal Care and Use Committee of the University of the Ryukyus.

INS Release Assay

INS release was measured by incubating the cells in Functionality/Viability Medium CMRL1066 (Mediatech). The cells were washed three times in PBS and incubated in the solution (Functionality/Viability Medium CMRL1066) with 2.8 mM D-glucose six times for 20 min each (total 2 h) to wash them. The cells were then incubated in the solution with 2.8 mM D-glucose for 2 h and then in the solution with 20 mM D-glucose for 2 h. The INS levels in the culture supernatants were measured using an Ultrasensitive Human Insulin ELISA kit (Mercodia).

Bisulfite Genomic Sequencing

Bisulfite treatment was performed using the CpGenome Turbo Bisulfite Modification Kit (Merck Millipore) according to the manufacturer's recommendations. The PCR primers are listed in Table S1. Amplified products were cloned using a Mighty TA-Cloning Kit (Takara Bio, Shiga, Japan). Ten randomly selected clones were sequenced with the M13 forward and reverse primers for each gene.

Microarrays

The total RNA from ESCs, iTS-P cells, or islets was labeled with biotin. Samples were hybridized using a GeneChip 3′IVT PLUS Reagent Kit (Affymetrix, Tokyo, Japan) and a GeneChip Hybridization, Wash and Stain Kit (Affymetrix) according to the manufacturer's protocol. Arrays were scanned using a GeneChip Scanner 3000 7G (Affymetrix). Data were analyzed using the Transcriptome Analysis Console (Affymetrix).

Statistical Analysis

The data are expressed as the mean ± SE. To compare the data among groups, we used a repeated-measures ANOVA test. Two groups were compared using the Student's t test. The differences between each group were considered significant if the p value was <0.05.

All methods were performed in accordance with the relevant guidelines and regulations.

SUPPLEMENTAL INFORMATION

Supplemental Information includes three figures and one table and can be found with this article online at https://doi.org/10.1016/j.omtm.2019.01.011.

AUTHOR CONTRIBUTIONS

H.N. designed the experiments, carried out most of the experimental work, and analyzed the data with the help of C.M.-S., Y.N., and T. Kinjo. N.K., I.S., M.W., A.M.J.S., and T. Kin provided materials and discussion. H.N. wrote the manuscript. All authors reviewed and critiqued the manuscript.

CONFLICTS OF INTEREST

The authors declare no competing interests.

ACKNOWLEDGMENTS

We thank Naomi Kakazu (University of the Ryukyus) for administrative assistance, and Maki Higa, Yuki Kawahira, and Saori Adaniya (University of the Ryukyus) for technical support. This work was supported in part by JSPS KAKENHI grants JP16H05404, JP16K10435, JP18K08545, JP15H04297, and JP16K15689; the Japan Agency for Medical Research and Development; the Okinawa Science and Technology Innovation System Construction Project; the Waksman Foundation of Japan, Inc; and The Naito Foundation.

REFERENCES

1. Bonner-Weir, S., Taneja, M., Weir, G.C., Tatarkiewicz, K., Song, K.H., Sharma, A., and O'Neil, J.J. (2000). In vitro cultivation of human islets from expanded ductal tissue. Proc. Natl. Acad. Sci. USA *97*, 7999–8004.

2. Heremans, Y., Van De Casteele, M., in't Veld, P., Gradwohl, G., Serup, P., Madsen, O., Pipeleers, D., and Heimberg, H. (2002). Recapitulation of embryonic neuroendocrine differentiation in adult human pancreatic duct cells expressing neurogenin 3. J. Cell Biol. *159*, 303–312.

3. Gao, R., Ustinov, J., Pulkkinen, M.A., Lundin, K., Korsgren, O., and Otonkoski, T. (2003). Characterization of endocrine progenitor cells and critical factors for their differentiation in human adult pancreatic cell culture. Diabetes *52*, 2007–2015.

4. Street, C.N., Lakey, J.R., Shapiro, A.M., Imes, S., Rajotte, R.V., Ryan, E.A., Lyon, J.G., Kin, T., Avila, J., Tsujimura, T., and Korbutt, G.S. (2004). Islet graft assessment in the Edmonton Protocol: implications for predicting long-term clinical outcome. Diabetes *53*, 3107–3114.

5. Yamamoto, T., Yamato, E., Taniguchi, H., Shimoda, M., Tashiro, F., Hosoi, M., Sato, T., Fujii, S., and Miyazaki, J.I. (2006). Stimulation of cAMP signalling allows isolation of clonal pancreatic precursor cells from adult mouse pancreas. Diabetologia *49*, 2359–2367.

6. Noguchi, H., Oishi, K., Ueda, M., Yukawa, H., Hayashi, S., Kobayashi, N., Levy, M.F., and Matusmoto, S. (2009). Establishment of mouse pancreatic stem cell line. Cell Transplant. *18*, 563–571.

7. Kuise, T., Noguchi, H., Saitoh, I., Kataoka, H.U., Watanabe, M., Noguchi, Y., and Fujiwara, T. (2013). Isolation efficiency of mouse pancreatic stem cells is age dependent. Cell Med. *5*, 69–73.

8. Noguchi, H., Naziruddin, B., Jackson, A., Shimoda, M., Ikemoto, T., Fujita, Y., Chujo, D., Takita, M., Kobayashi, N., Onaca, N., et al. (2010). Characterization of human pancreatic progenitor cells. Cell Transplant. *19*, 879–886.

9. Takahashi, K., and Yamanaka, S. (2006). Induction of pluripotent stem cells from mouse embryonic and adult fibroblast cultures by defined factors. Cell *126*, 663–676.

10. Takahashi, K., Tanabe, K., Ohnuki, M., Narita, M., Ichisaka, T., Tomoda, K., and Yamanaka, S. (2007). Induction of pluripotent stem cells from adult human fibroblasts by defined factors. Cell *131*, 861–872.

11. Yu, J., Vodyanik, M.A., Smuga-Otto, K., Antosiewicz-Bourget, J., Frane, J.L., Tian, S., Nie, J., Jonsdottir, G.A., Ruotti, V., Stewart, R., et al. (2007). Induced pluripotent stem cell lines derived from human somatic cells. Science *318*, 1917–1920.

12. Park, I.H., Zhao, R., West, J.A., Yabuuchi, A., Huo, H., Ince, T.A., Lerou, P.H., Lensch, M.W., and Daley, G.Q. (2008). Reprogramming of human somatic cells to pluripotency with defined factors. Nature *451*, 141–146.

13. Maherali, N., Sridharan, R., Xie, W., Utikal, J., Eminli, S., Arnold, K., Stadtfeld, M., Yachechko, R., Tchieu, J., Jaenisch, R., et al. (2007). Directly reprogrammed fibroblasts show global epigenetic remodeling and widespread tissue contribution. Cell Stem Cell *1*, 55–70.

14. Okita, K., Ichisaka, T., and Yamanaka, S. (2007). Generation of germline-competent induced pluripotent stem cells. Nature *448*, 313–317.

15. Wernig, M., Meissner, A., Foreman, R., Brambrink, T., Ku, M., Hochedlinger, K., Bernstein, B.E., and Jaenisch, R. (2007). In vitro reprogramming of fibroblasts into a pluripotent ES-cell-like state. Nature *448*, 318–324.

16. Okita, K., Nakagawa, M., Hyenjong, H., Ichisaka, T., and Yamanaka, S. (2008). Generation of mouse induced pluripotent stem cells without viral vectors. Science *322*, 949–953.

17. Okita, K., Matsumura, Y., Sato, Y., Okada, A., Morizane, A., Okamoto, S., Hong, H., Nakagawa, M., Tanabe, K., Tezuka, K., et al. (2011). A more efficient method to generate integration-free human iPS cells. Nat. Methods *8*, 409–412.

18. Yu, J., Hu, K., Smuga-Otto, K., Tian, S., Stewart, R., Slukvin, I.I., and Thomson, J.A. (2009). Human induced pluripotent stem cells free of vector and transgene sequences. Science *324*, 797–801.

19. Stadtfeld, M., Nagaya, M., Utikal, J., Weir, G., and Hochedlinger, K. (2008). Induced pluripotent stem cells generated without viral integration. Science *322*, 945–949.

20. Yoshioka, N., Gros, E., Li, H.R., Kumar, S., Deacon, D.C., Maron, C., Muotri, A.R., Chi, N.C., Fu, X.D., Yu, B.D., and Dowdy, S.F. (2013). Efficient generation of human iPSCs by a synthetic self-replicative RNA. Cell Stem Cell *13*, 246–254.

21. Noguchi, H., Saitoh, I., Tsugata, T., Kataoka, H., Watanabe, M., and Noguchi, Y. (2015). Induction of tissue-specific stem cells by reprogramming factors, and tissue-specific selection. Cell Death Differ. *22*, 145–155.

22. Saitoh, I., Sato, M., Soda, M., Inada, E., Iwase, Y., Murakami, T., Ohshima, H., Hayasaki, H., and Noguchi, H. (2016). Tissue-specific stem cells obtained by reprogramming of non-obese diabetic (NOD) mouse-derived pancreatic cells confer insulin production in response to glucose. PLoS ONE *11*, e0163580.

23. Miyagi-Shiohira, C., Nakashima, Y., Kobayashi, N., Saitoh, I., Watanabe, M., and Noguchi, H. (2018). Characterization of induced tissue-specific stem cells from pancreas by a synthetic self-replicative RNA. Sci. Rep. *8*, 12341.

24. Noguchi, H., Miyagi-Shiohira, C., and Nakashima, Y. (2018). Induced tissue-specific stem cells and epigenetic memory in induced pluripotent stem cells. Int. J. Mol. Sci. *19*, E930.

25. Kim, K., Doi, A., Wen, B., Ng, K., Zhao, R., Cahan, P., Kim, J., Aryee, M.J., Ji, H., Ehrlich, L.I., et al. (2010). Epigenetic memory in induced pluripotent stem cells. Nature *467*, 285–290.

26. Polo, J.M., Liu, S., Figueroa, M.E., Kulalert, W., Eminli, S., Tan, K.Y., Apostolou, E., Stadtfeld, M., Li, Y., Shioda, T., et al. (2010). Cell type of origin influences the molecular and functional properties of mouse induced pluripotent stem cells. Nat. Biotechnol. *28*, 848–855.

27. Doi, A., Park, I.H., Wen, B., Murakami, P., Aryee, M.J., Irizarry, R., Herb, B., Ladd-Acosta, C., Rho, J., Loewer, S., et al. (2009). Differential methylation of tissue- and cancer-specific CpG island shores distinguishes human induced pluripotent stem cells, embryonic stem cells and fibroblasts. Nat. Genet. *41*, 1350–1353.

28. Lister, R., Pelizzola, M., Kida, Y.S., Hawkins, R.D., Nery, J.R., Hon, G., Antosiewicz-Bourget, J., O'Malley, R., Castanon, R., Klugman, S., et al. (2011). Hotspots of aberrant epigenomic reprogramming in human induced pluripotent stem cells. Nature *471*, 68–73.

29. Ohi, Y., Qin, H., Hong, C., Blouin, L., Polo, J.M., Guo, T., Qi, Z., Downey, S.L., Manos, P.D., Rossi, D.J., et al. (2011). Incomplete DNA methylation underlies a transcriptional memory of somatic cells in human iPS cells. Nat. Cell Biol. *13*, 541–549.

30. Bar-Nur, O., Russ, H.A., Efrat, S., and Benvenisty, N. (2011). Epigenetic memory and preferential lineage-specific differentiation in induced pluripotent stem cells derived from human pancreatic islet beta cells. Cell Stem Cell *9*, 17–23.

31. D'Amour, K.A., Bang, A.G., Eliazer, S., Kelly, O.G., Agulnick, A.D., Smart, N.G., Moorman, M.A., Kroon, E., Carpenter, M.K., and Baetge, E.E. (2006). Production of pancreatic hormone-expressing endocrine cells from human embryonic stem cells. Nat. Biotechnol. *24*, 1392–1401.

32. Kroon, E., Martinson, L.A., Kadoya, K., Bang, A.G., Kelly, O.G., Eliazer, S., Young, H., Richardson, M., Smart, N.G., Cunningham, J., et al. (2008). Pancreatic endoderm derived from human embryonic stem cells generates glucose-responsive insulin-secreting cells in vivo. Nat. Biotechnol. *26*, 443–452.

33. Cheng, X., Ying, L., Lu, L., Galvão, A.M., Mills, J.A., Lin, H.C., Kotton, D.N., Shen, S.S., Nostro, M.C., Choi, J.K., et al. (2012). Self-renewing endodermal progenitor lines generated from human pluripotent stem cells. Cell Stem Cell *10*, 371–384.

34. Greber, B., Coulon, P., Zhang, M., Moritz, S., Frank, S., Müller-Molina, A.J., Araúzo-Bravo, M.J., Han, D.W., Pape, H.C., and Schöler, H.R. (2011). FGF signalling inhibits neural induction in human embryonic stem cells. EMBO J. *30*, 4874–4884.

35. Panciera, T., Azzolin, L., Fujimura, A., Di Biagio, D., Frasson, C., Bresolin, S., Soligo, S., Basso, G., Bicciato, S., Rosato, A., et al. (2016). Induction of expandable tissue-specific stem/progenitor cells through transient expression of YAP/TAZ. Cell Stem Cell *19*, 725–737.

36. Wang, Y., Qin, J., Wang, S., Zhang, W., Duan, J., Zhang, J., Wang, X., Yan, F., Chang, M., Liu, X., et al. (2016). Conversion of human gastric epithelial cells to multipotent endodermal progenitors using defined small molecules. Cell Stem Cell *19*, 449–461.

37. Shapiro, A.M., Lakey, J.R., Ryan, E.A., Korbutt, G.S., Toth, E., Warnock, G.L., Kneteman, N.M., and Rajotte, R.V. (2000). Islet transplantation in seven patients with type 1 diabetes mellitus using a glucocorticoid-free immunosuppressive regimen. N. Engl. J. Med. *343*, 230–238.

OMTM, Volume *13*

Supplemental Information

Induction of Expandable Tissue-Specific Progenitor Cells from Human Pancreatic Tissue through Transient Expression of Defined Factors

Hirofumi Noguchi, Chika Miyagi-Shiohira, Yoshiki Nakashima, Takao Kinjo, Naoya Kobayashi, Issei Saitoh, Masami Watanabe, A. M. James Shapiro, and Tatsuya Kin

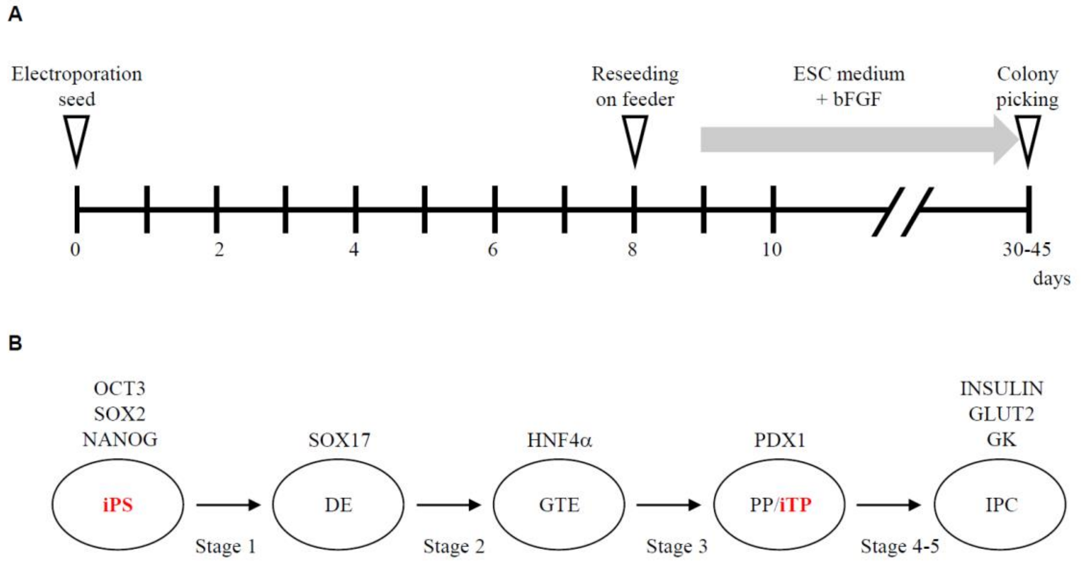

Supplemental Fig 1 Noguchi et al.

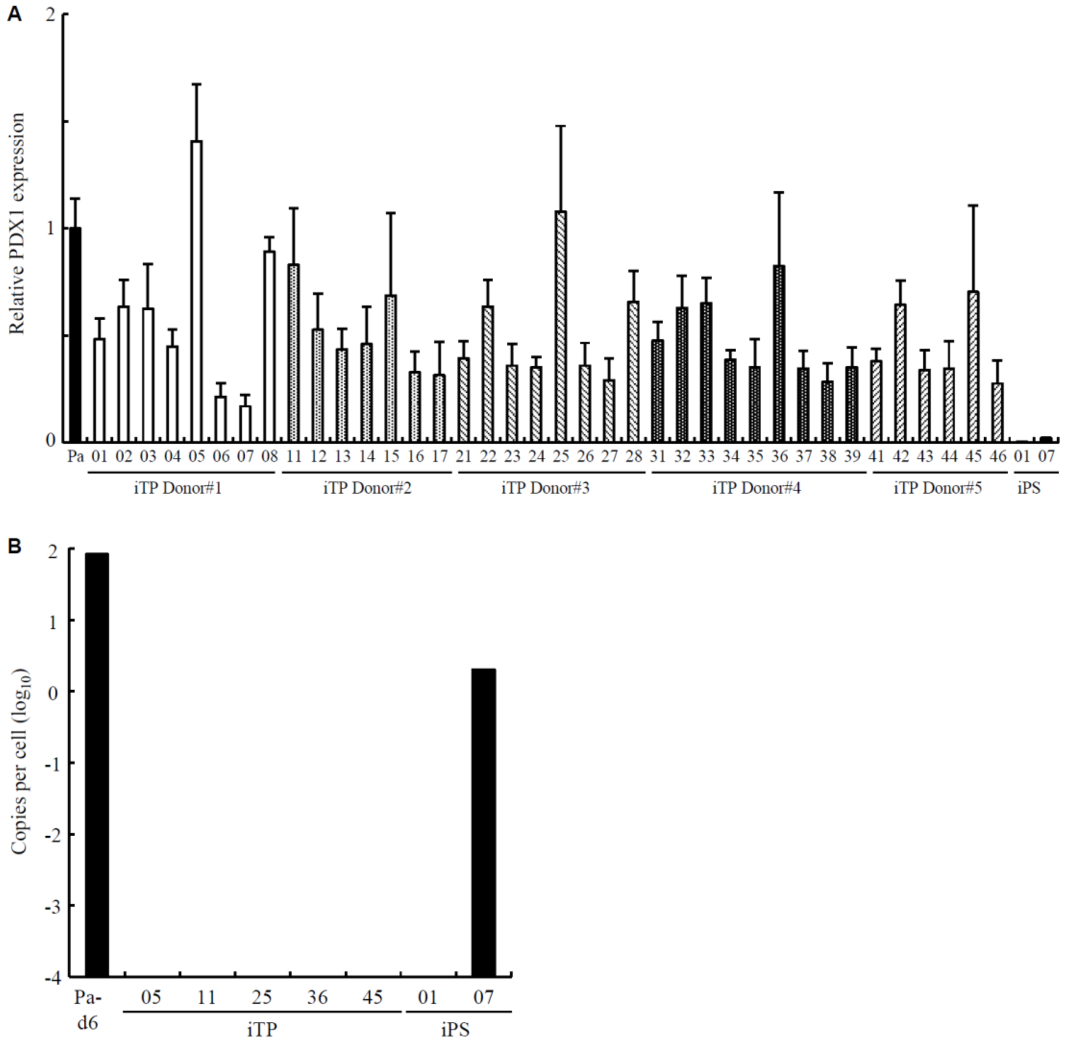

Supplemental Fig 2 Noguchi et al.

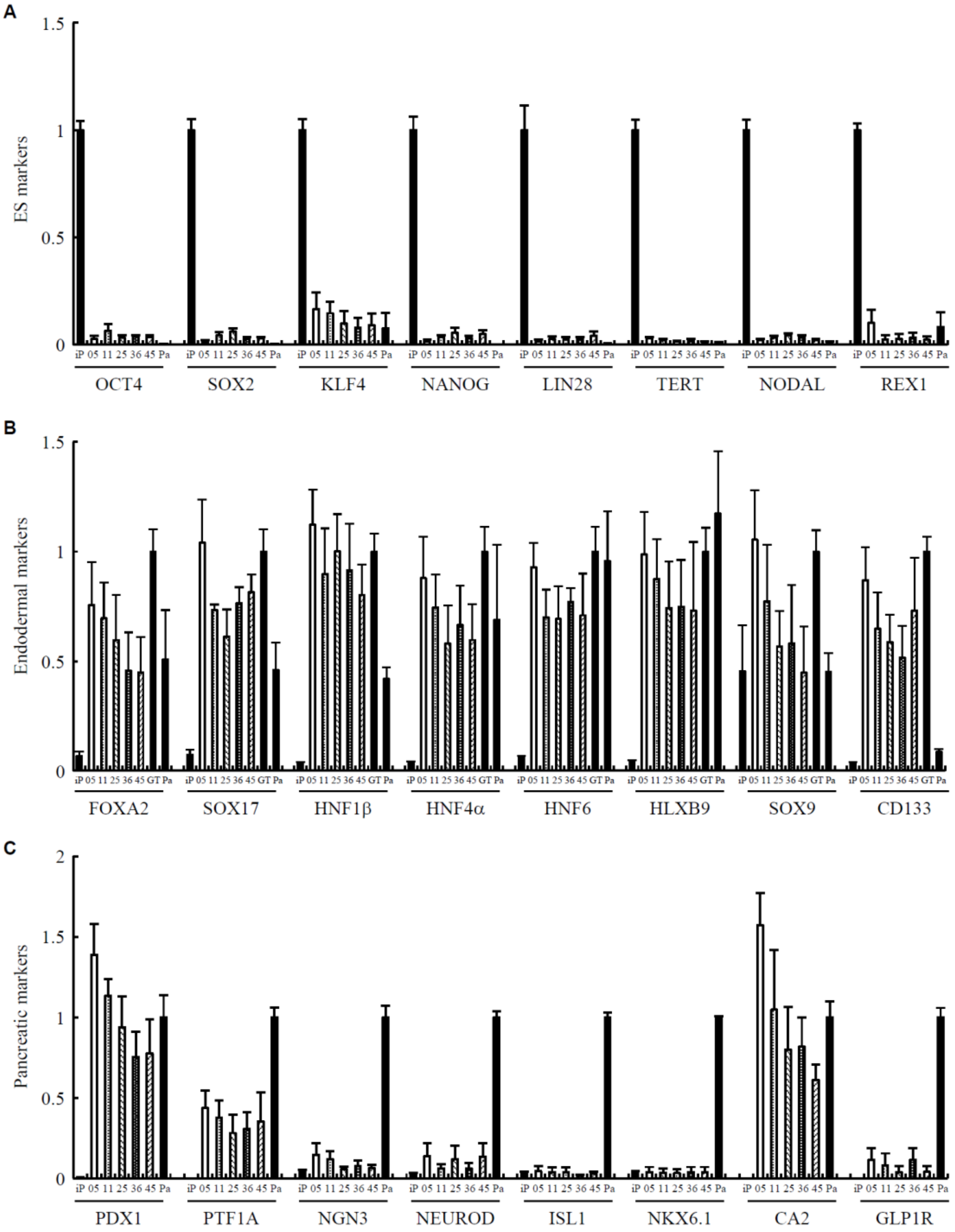

Supplemental Fig 3 Noguchi et al.

SUPPLEMENTAL FIGURE LEGENDS

Supplemental Figure 1. Time schedules for the generation of human iTP cells from pancreatic tissue and differentiation protocol of iPS/iTP cells. (A) Time schedules for the induction of iTP cells. Human pancreatic tissue (1×10^5 cells) was electroporated with episomal plasmid vectors expressing four (OCT3/4, SOX2, KLF4, c-MYC) or six (OCT3/4, p53 shRNA, SOX2, KLF4, L-MYC, and LIN28) reprogramming factors and plated on day 0. Cells were reseeded on feeder on day 8. Cells were then cultured in ESC medium plus bFGF. (B) A schematic representation of the stepwise differentiation of iPS/iTP cells to insulin-producing cells. The cells of the definitive endoderm (DE) express SOX17; cells of the gut tube endoderm (GTE) express HNF4α; pancreatic progenitors (PP) express PDX1; and insulin-producing cells (IPC) express INSULIN, GLUT2, and GLUCOKINASE (GK).

Supplemental Figure 2. Evaluation of human iTP cells from pancreatic tissue

(A) Quantitative RT-PCR analysis of PDX1, a marker of pancreatic stem/progenitor cells, in iTP and iPS cells. Thirty-eight iTP clones and two iPS clones were evaluated for PDX1 expression using quantitative RT-PCR. The data are expressed as the PDX1-to-GAPDH ratio, with the ratio of pancreatic tissue arbitrarily set to 1 (n = 5). Error bars

represent the standard error. (B) Copy numbers of episomal plasmid vectors in iTP and iPS clones. Pancreatic tissue 6 days after electroporation of plasmid vectors expressing six reprogramming factors were analyzed (Pa-d6) as a positive control.

Supplemental Figure 3. Quantitative RT-PCR analysis of human iTP cells for marker genes of ES and endodermal/pancreatic cells.

(A) qRT-PCR analysis of ES cell marker genes in human iTP05, 11, 25, 36, and 45 cells. iPS cells served as a control. (B) qRT-PCR analysis of endodermal cell marker genes in human iTP05, 11, 25, 36, and 45 cells. GTE cells were used as a control. (C) q RT-PCR analysis of pancreatic cell marker genes in human iTP05, 11, 25, 36, and 45 cells. Pancreatic cells (islets >80%) cells were used as a control. iP, iPS cells; 05/ 11/ 25/ 36/ 45, iTP cells; Pa, pancreatic cells (islets >80%); GT, GTE cells. The data are expressed as the gene-to-GAPDH ratio, with that of the control cells arbitrarily set to 1 (n = 4). The error bars represent the standard error.

Supplemental Table 1. Primers

	Forward	Reverse
OCT3/4 p	gaggttggagtagaaggattgttttggttt	cccccctaacccatcacctccaccacctaa
NANOG p	tggttaggttggttttaaatttttg	aacccacccttataaattctcaatta
PDX1 p	ctcagctgagagagaaaattggaa	actaatctcaaaaaaaacccac
